# Comparison of Oxidative Properties, Light Absorbance, and Total and Elemental Mass Concentration of Ambient PM_2.5_ Collected at 20 European Sites

**DOI:** 10.1289/ehp.8584

**Published:** 2005-12-29

**Authors:** Nino Künzli, Ian S. Mudway, Thomas Götschi, Tingming Shi, Frank J. Kelly, Sarah Cook, Peter Burney, Bertil Forsberg, James W. Gauderman, Marianne E. Hazenkamp, Joachim Heinrich, Deborah Jarvis, Dan Norbäck, Felix Payo-Losa, Albino Poli, Jordi Sunyer, Paul J.A. Borm

**Affiliations:** 1 Working Group Air Pollution, European Community Respiratory Health Survey, London, United Kingdom, and Barcelona, Spain; 2 Keck School of Medicine, University of Southern California, Los Angeles, California, USA; 3 Lung Biology, Pharmaceutical Sciences Research Division, King’s College, London, United Kingdom; 4 Institut fuer Umweltmedizinische Forschung, Duesseldorf, Germany; 5 Hubei Provincial Center for Disease Control and Prevention, Hubei, People’s Republic of China; 6 Department of Public Health Sciences, Kings College, London, United Kingdom; 7 Department of Public Health and Clinical Medicine, Umeå University, Umeå, Sweden; 8 Institute of Social and Preventive Medicine, University of Basel, Basel, Switzerland; 9 GSF–National Research Centre for Environment and Health, Institute of Epidemiology, Neuherberg, Germany; 10 Department of Occupational and Environmental Medicine, University Hospital Uppsala, Uppsala, Sweden; 11 Hospital Central de Asturias, Oviedo, Spain; 12 Department of Medicine and Public Health, University of Verona, Verona, Italy; 13 Institut Municipal de Investigacio Medica, Barcelona, Spain; 14 Centre of Expertise in Life Sciences, Zuyd University, Heerlen, the Netherlands

**Keywords:** air pollution, antioxidant depletion, ascorbate, black smoke, elemental analysis, fine particle, glutathione, hydroxy radical formation, oxidative stress, reactive oxidant species, reflectance

## Abstract

**Objective:**

It has been proposed that the redox activity of particles may represent a major determinant of their toxicity. We measured the *in vitro* ability of ambient fine particles [particulate matter with aerodynamic diameters ≤2.5 μm (PM_2.5_)] to form hydroxyl radicals (^•^OH) in an oxidant environment, as well as to deplete physiologic antioxidants (ascorbic acid, glutathione) in the naturally reducing environment of the respiratory tract lining fluid (RTLF). The objective was to examine how these toxicologically relevant measures were related to other PM characteristics, such as total and elemental mass concentration and light absorbance.

**Design:**

Gravimetric PM_2.5_ samples (*n* = 716) collected over 1 year from 20 centers participating in the European Community Respiratory Health Survey were available. Light absorbance of these filters was measured with reflectometry. PM suspensions were recovered from filters by vortexing and sonication before dilution to a standard concentration. The oxidative activity of these particle suspensions was then assessed by measuring their ability to generate ^•^OH in the presence of hydrogen peroxide, using electron spin resonance and 5,5-dimethyl-1-pyrroline-*N*-oxide as spin trap, or by establishing their capacity to deplete antioxidants from a synthetic model of the RTLF.

**Results and Conclusion:**

PM oxidative activity varied significantly among European sampling sites. Correlations between oxidative activity and all other characteristics of PM were low, both within centers (temporal correlation) and across communities (annual mean). Thus, no single surrogate measure of PM redox activity could be identified. Because these novel measures are suggested to reflect crucial biologic mechanisms of PM, their use may be pertinent in epidemiologic studies. Therefore, it is important to define the appropriate methods to determine oxidative activity of PM.

Epidemiologic studies have observed significant associations between the mass concentration of various size fractions of ambient particulate matter (PM) and cardiorespiratory health (Brunekreef and Holgate 2002). Although the general pattern of results appears sufficiently consistent to conclude that PM does contribute to the adverse health effects of ambient air pollution, heterogeneity in the estimated size of these health effects has been described across studies, even where identical methods were employed (Bell et al. 2004; Janssen et al. 2002; Katsouyanni et al. 2001; Samet et al. 2000; Zanobetti et al. 2002). Apart from random variation and differences across study populations, such discrepancies may originate from the use of imperfect indicators of exposure. Most studies use PM mass concentration. This measurement ignores sources and constituents and therefore does not give a comprehensive measure of biologic activity. The validity of using this exposure marker to assess the health effects of ambient PM largely depends on the correlation between PM mass concentration and the toxicologically relevant feature(s) of these particles, which may vary regionally.

There is increasing scientific support for theories proposing that oxidative and nitrosative stress represent a primary pathway leading to the respiratory and systemic inflammatory responses associated with PM exposure (Gilliland et al. 1999; Li et al. 2002, 2003; Nel 2005; Nel et al. 2001; Xia et al. 2004). As recently reviewed (Donaldson et al. 2005; Knaapen et al. 2004), the capacity of PM to elicit oxidative stress reflects both the oxidant-generating properties of particles and their ability to stimulate cellular generation of reactive oxidant species (Pourazar et al. 2005; Prahalad et al. 1999). Inflammation as well as the oxidative properties of PM may also modify the permeability of the lung, thus directly affecting the translocation of nanoparticles (Heckel et al. 2004; Meiring et al. 2005).

From a mechanistic point of view, it is appealing to use biologically relevant properties of PM to characterize “exposure” in epidemiologic studies, rather than imperfect surrogates such as the mass concentration of various size fractions, surface area, content of metals, organics, or other markers. We measured the oxidative capacity of ambient fine particles [PM with aerodynamic diameters ≤2.5 μm (PM_2.5_)] by measuring their ability to generate hydroxyl radicals (^•^OH) in the presence of hydrogen peroxide (H_2_O_2_). Hydroxyl radical formation plays an important role in the induction of oxidative stress and inflammatory responses in the lung (Donaldson et al. 1997; Li et al. 1996). The formation of ^•^OH by PM has been associated *in vitro* with premutagenic DNA adducts and oxidative DNA damage (Knaapen et al. 2002; Shi et al. 2003a, 2003b) as well as human inflammatory responses after bronchial instillation (Schaumann et al. 2004). Thus, the formation of ^•^OH by PM may be a relevant marker for the initiation of various health effects (Borm et al. 2004). Because ^•^OH formation may be driven by an array of PM characteristics or constituents such as surface area, size, transition metal, polycyclic aromatic hydrocarbons, or quinone content (Knaapen et al. 2002, 2004; Nel 2005), its direct measurement is appealing as a way of integrating these various parameters into a single measure of toxicity.

In addition to the generation of ^•^OH, we examined the capacity of ambient PM_2.5_ to deplete antioxidants within the respiratory tract lining fluid (RTLF), specifically the major low-molecular-weight antioxidants, ascorbic acid (AA), uric acid, and glutathione (GSH) (Kelly et al. 1996). Pulmonary antioxidants have been shown to represent an important defense against PM-induced oxidative damage (Anseth et al. 2005; Greenwell et al. 2003; Kelly et al. 1996). The extent to which these antioxidants are depleted by PM reflects a direct measure of their oxidative activity. This method complements the ^•^OH generation approach by examining oxidation reactions in the absence of H_2_O_2_, which we interpret as reflecting a healthy lung scenario as opposed to a disease/inflamed lung where H_2_O_2_ concentrations are known to be elevated (Zielinski et al. 1999).

We applied these novel methods to a large sample of ambient PM_2.5_ collected over 1 year at 20 monitoring sites in 19 cities participating in the European Community Respiratory Health Survey II (ECRHS II) (European Community Respiratory Health Group 2002; Hazenkamp-von Arx et al. 2003). We compared the oxidative properties of these samples with the PM_2.5_ mass concentration, light absorption (hereafter referred to as absorbance) as a proxy of elemental carbon, and the mass concentration of selected chemical elements (Götschi et al. 2005). We chose elements that are known to participate directly or indirectly in redox chemistry (aluminum, arsenic, copper, iron, manganese, lead, titanium, vanadium, zinc, sulfur) as an optimal marker for long-range urban background pollution, and silicon as a marker of crustal PM.

Our comparison focuses on three criteria relevant to epidemiologic studies where measurements are taken at one or more locations to characterize exposure to pollutants from outdoor origin. First, we describe the variation of all PM characteristics throughout the year (temporal or seasonal variation). This has implications in studies investigating acute effects of the oxidative properties where high day-to-day variation is an advantage. Second, we assess the correlation of the oxidative properties with all other characteristics at each of the 20 locations. This demonstrates the degree to which temporal changes of other, more readily available measures of PM may serve as surrogates for the temporal variation of oxidative properties seen at the same location. Third, we compare the annual means of all characteristics across the 20 centers. This perspective is of relevance in cross-community comparisons where associations between the communitywide air quality and (adjusted) health outcomes are investigated. If cross-community correlations between oxidative properties and some other PM characteristic were high, the latter may be used as a surrogate in the absence of measurements of ^•^OH formation or antioxidant depletion, even in case of poor temporal correlations within each city. This third criterion is of particular interest for the use of these data for health effect analyses in ECRHS II.

Concentrations and distributions of mass, light absorbance, and elemental contents of these PM have been published for the full PM_2.5_ data collected in ECRHS (Götschi et al. 2005; Hazenkamp-von Arx et al. 2004).

## Materials and Methods

### Sampling and characterization of PM_2.5_.

ECRHS II follows up on the populations assessed cross-sectionally in 1990–1993 (Burney et al. 1994; European Community Respiratory Health Group 2002; Janson et al. 2001). In total, 21 centers in 20 cities participated in the air pollution module implemented in ECRHS II. The lack of standardized air pollution monitoring networks across Europe required an assessment of the long-term average air quality in each center. The measurements started between June and December 2000 and lasted at least 12 months. The primary focus was the gravimetric sampling of PM_2.5_. The methods and the main results have been published elsewhere (Hazenkamp-von Arx et al. 2003, 2004). In brief, a standardized protocol was implemented, using identical equipment [Basel-Sampler (BGI Inc., Waltham, MA, USA); Teflon filters] and a single weighing laboratory. The sampling schedule was designed to sample 7 days over a 2-week period during each month, yielding 84 days over a 1-year period. Weekday samples were exposed 24 hr, whereas weekends were captured on single filters exposed for 48 hr (in total 72 filters/center).

As described by Götschi et al. (2005), PM_2.5_ filter samples were analyzed for 26 different chemical elements, using energy-dispersive X-ray fluorescence spectrometry (ED-XRF). This nondestructive method was applied by the same laboratory with the same methods as in the EXPOLIS study (Mathys et al. 2001). Fifteen elements were detected reliably and in most centers. Carbon could not be detected in the elemental analysis, but light absorbance of the filters was measured as a surrogate for elemental carbon. We used the same standard method and device (Reflectometer EEL model 43; Diffusion Systems Ltd., London, U.K.) as described before (Götschi et al. 2002). PM_2.5_ and elemental concentrations are reported as mass concentrations per volume of air. Absorbance is reported as absorption coefficient, which is comparable with mass concentration per air volume.

For the analyses of oxidative properties, every second filter (*n* = 716) underwent a standardized extraction in 1 mL metal-free water, with the resultant suspension then diluted to a concentration of 200 μg/mL. The procedure has been described elsewhere (Shi et al. 2003b) but is detailed here for interpretation of the data. To prepare PM suspensions from Teflon filters, the support ring was removed, and the filter was placed into double-distilled water and agitated (5 min) before sonication in a water bath (5 min). After sonication, a further 5 min agitation was performed. Blank filters were treated in the same way and used as controls in all experiments.

Generation of ^•^OH by particle suspensions was studied in the presence of H_2_O_2_ and the spin trap 5,5-dimethyl-1-pyrroline-*N*-oxide (DMPO). For ^•^OH measurement, 50 μL of the particle suspension was mixed with 50 μL H_2_O_2_ [0.5 M in phosphate-buffered saline (PBS)] and 100 μL DMPO (0.05 M in PBS). The mixture was incubated in the dark and shaken continuously at 37°C before being filtered through a 0.1-μm-pore filter (Acrodisc 25-mm syringe filter; Pall Gelman Laboratory, Ann Arbor, MI, USA). The clear filtrate was transferred immediately to a 100-μL glass capillary and measured with a Miniscope MS100 EPR spectrometer (Magnettech, Berlin, Germany) under standard conditions. Quantification was carried out as the sum of total amplitudes of DMPO–^•^OH quartet signal, and the outcome is expressed as the total amplitude in arbitrary units, related to the same instrumental settings. To quantify the weight of the particulates recovered from the filters, we used two methods to estimate the concentration per milliliter: comparative turbidometry at 405 nm against a carbon black standard (Huber 990, 260 nm; H. Haeffner & Co., Ltd., Chepstow, UK), and weighing of a subset of 32 Teflon filters before and after removal of particles and weighing the filter after drying. The first method is based on the comparison of the “blackness” of the particle suspension to standard carbon black (260 nm) by spectroscopic absorption at 405 nm. Although part of the PM is water soluble and maybe seen as an extract, our comparative experiments using gravimetric analysis of PM before and after filter extraction confirmed the correlation between turbidometry and gravimetric analysis. It also demonstrated that average recovery for 30 PM_2.5_ samples using this procedure was 88% (*y* = 1.22 × *x; r*^2^ = 0.724; *n* = 30).

Further, we assessed the particles’ ability to deplete the antioxidants AA, urate, and GSH in an established model of lung-lining fluid (Zielinski et al. 1999). To yield sufficient material for these analyses, suspensions of filters from each 2-month period had to be pooled, reducing the sample size for the antioxidant depletion measurements to six suspensions per location. Each pool contained the PM suspended from six filters (see above: one filter for each weekday and one filter for the weekend, for a 2-month period). The pooled suspensions (*n* = 114) were diluted to 50 μg/mL and incubated in a composite antioxidant solution (pH 7.0, 37°C) containing 200 μM of AA, urate, and GSH for 4-hr with gentle mixing. Particle-free 0- and 4-hr antioxidant controls, as well as 4-hr positive (residual oil fly ash) and negative (carbon black) particle controls, were run in parallel to quantify autooxidative losses and permit standardization between batches of samples. Then the 4-hr incubation samples were centrifuged for 1 hr at 13,000 rpm (4°C). Aliquots of the resultant supernatants were then either acidified with metaphosphoric acid to a final concentration of 5% (wt/vol) for AA and urate measurement or diluted into 100 mM phosphate buffer for the determination of reduced GSH. Determination of AA, urate, and reduced GSH has been described in detail previously (Mudway et al. 2004; Zielinski et al. 1999). In this article, we focus on AA and GSH only because PM did not deplete urate, consistent with earlier observations (Mudway et al. 2004; Zielinski et al. 1999). ^•^OH generation and depletion of antioxidants are reported as concentrations of arbitrary units per standard mass of particles.

### Statistical analyses.

We show the temporal variation of PM properties using the coefficient of variation (CV) across all filters at each location. The CV is defined as the standard deviation divided by the mean of all single measurements taken at a given location. For each monitoring location, we also present the filter-to-filter Spearman rank correlation for ^•^OH generation versus all other simultaneously measured PM properties. For AA and GSH we did not derive center-specific correlations because we had only six samples available per center. In the last step, we present Pearson correlations of the annual means across the centers to discuss the use of other constituents as proxies for ^•^OH, AA, and GSH annual means.

## Results

### Temporal variation.

Table 1 presents the temporal variability of all measures in each location. A pattern common to all centers reveals that ^•^OH formation and AA varied the least, whereas Cu and Pb exerted the highest temporal variation over the year. The antioxidant depletions are based on the pooled filters; thus, the values of temporal variability are not directly comparable with the others. The identical aggregation procedure for all markers indicated that AA (CV = 0.20, on average) and ^•^OH formation (0.26) showed less temporal variability across the six bimonthly values than did PM_2.5_ mass (0.33), whereas GSH was more variable (0.71). It should be noted, however, that the magnitude of GSH loss was substantially less than was seen for AA. We emphasize that annual means of mass concentrations provided in Table 1 are less precise and slightly different from the annual means published previously (Götschi et al. 2005; Hazenkamp-von Arx et al. 2004) because redox activity has been determined in only half the ECRHS filter material.

### Temporal correlation between PM characteristics at the same location.

As shown in Table 2, daily ambient concentrations of the available PM constituents were, in most cases and in all cities, poorly correlated with ^•^OH formation. Correlations with ^•^OH generation were not only low but also heterogeneous across locations. Spearman correlations between ^•^OH generation and PM_2.5_ mass concentration ranged between –0.49 and 0.61. Although in Erfurt or Oviedo elemental Fe associated rather well with ^•^OH generation, this was not the case in other centers.

### Cross-community comparison.

**Figure 1:**
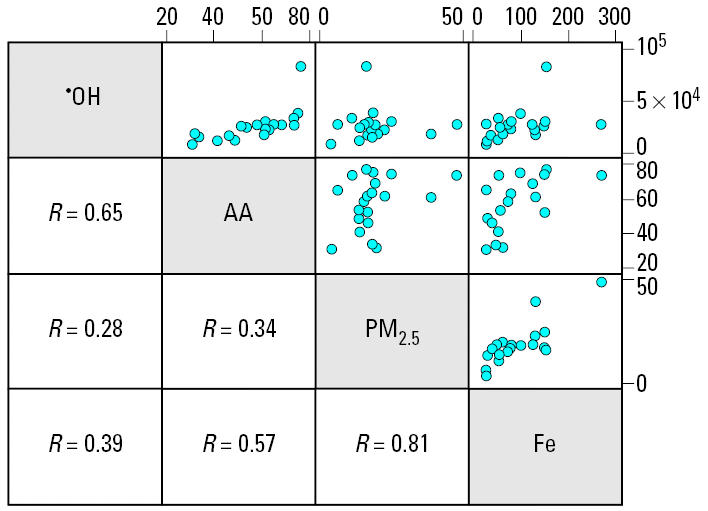
Scatter plot and Pearson correlation of annual mean ^•^OH, AA depletion rates, PM_2.5_ total mass, and Fe mass concentration (units as in Table 1).

As previously reported for total and elemental mass concentration as well as absorbance (Götschi et al. 2005; Hazenkamp-von Arx et al. 2004), oxidative activity of PM_2.5_ also varied substantially across these European cities. As shown in the last row of Table 1, the 10-fold contrast in the annual average ^•^OH formation is, however, smaller than for most elements and for light absorbance. Correlations between oxidative properties and all other characteristics were mostly weak. The best proxies of the capacity of PM to deplete AA were annual mean Cu (*r* = 0.60), Fe (0.59), and Zn (0.50) content, as well as filter absorbance (0.49). Figure 1 demonstrates that a few outliers did not drive the lack of correlation. The capacity of PM to deplete GSH was best correlated with Al (0.55) and Cu (0.49 annual mean PM concentrations) (Table 3).

## Discussion

This is the first large-scale investigation of PM_2.5_ oxidant activity to assess the use of ^•^OH formation as well as antioxidant depletion. In light of the novelty of this undertaking, we discuss three main questions related to the further development and use of these redox characteristics in air pollution research: their biologic relevance, methodologic issues of validity, and their application in epidemiologic studies.

### Is oxidant activity of PM relevant?

This study was based on the assumption that PM-induced oxidative stress is central to the observed toxicity *in vivo* and underlies many of the health effects observed in exposed populations (Donaldson et al. 2003). Support for this contention, associating particle oxidative components with health effects, has now been published by numerous groups (Donaldson et al. 1996, 2003, 2005; Gilliland et al. 1999; Nel 2005; Prahalad et al. 2000; Shi et al. 2003b; Zielinski et al. 1999). We used two different measurements of PM-induced oxidative stress: ^•^OH formation in an oxidant environment (in the presence of H_2_O_2_) and antioxidant depletion in a synthetic RTLF, reflecting the normal reducing environment at the air–lung interface. In this study, both of these assays were highly correlated, demonstrating that particle suspensions recovered from filters contain components able to cause oxidative stress under very different conditions. Both methods are therefore appealing in investigating PM-related health effects mediated through redox mechanisms. Hence, the capacity of PM to stimulate the production of ^•^OH in the presence of H_2_O_2_ can be seen as indicative of reactions likely to occur in a diseased/inflamed lung, whereas the RTLF, containing AA, urate, and GSH, is more reflective of a healthy lung scenario (Shi et al. 2003a; Zielinski et al. 1999). The importance of these radical-generating processes has been shown in studies demonstrating DNA oxidation in response to PM-induced production of ^•^OH (Shi et al. 2003b; van Maanen et al. 1999).

Both the PM-stimulated formation of ^•^OH and the loss of RTLF antioxidants can be attributed to the transition metal content of PM. Several transition metals [Fe, Cu, chromium (Cr), Ti, nickel (Ni), cobalt (Co), and V] will react with H_2_O_2_ to form ^•^OH via the Haber-Weiss reaction (Halliwell 1999), whereas the loss of AA and GSH is caused both by their reduction of oxidized metal ions [Fe(III), Cu(II), Cr(VI)] and by the subsequent formation of superoxide (Buettner et al. 1996). Notably, under aerobic conditions, at neutral pH, Ni, Ti, and Co will not act as catalysts for AA oxidation (Fridman et al. 1979) such that the two assays of oxidative activity are sensitive to slightly different panels of metals. In addition, AA will undergo catalytic oxidation in the presence of quinone compounds (Roginsky et al. 1999), such that the antioxidant depletion assay is sensitive to both metal and quinone/hydroquinone components of PM. In contrast, quinones do not yield ^•^OH in the electron paramagnetic resonance–based assay. The strong correlation between the capacity of PM to generate ^•^OH and deplete AA would therefore suggest that PM quinone content does not contribute significantly to the oxidative activity in these archive filter PM samples, and that redox active metal components such as Fe and Cu are more important determinants of the observed activity. We therefore conclude that the use of redox activity of PM may greatly advance our understanding of health effects of air pollution and that the use of complementary methods may help identify those components driving the observed activity.

### Is oxidative activity correctly measured?

Although we have argued that the capacity of PM samples to cause oxidation reactions is an important determinant of their biologic activity, this assumption holds true only if the PM samples extracted from filters are representative of those breathed in ambient air. Particles collected on Teflon filters or other substrates and stored before sonication may not be a perfect model for PM as encountered by the lung under real-life conditions because of the impact of sonication and aging processes on the PM components. Volatile constituents of PM such as organic fractions that represent a potential source of redox activity in fresh PM may not be fully captured onto filters, and extraction procedures into water may not fully extract those present (Li et al. 2002, 2003; Xia et al. 2004). We have previously shown that ambient PM extracted into water or methanol has equivalent activity (data not shown), suggesting this may not be a significant problem, although clearly this will depend on the composition of the sampled PM. Mass recovery for PM_2.5_ in this study was high but not complete (88%), and this may have confounded our data, because ultrafine particles are less well recovered from these filters than larger particles.

Samplers have been developed to collect fresh particles, but measurements are labor intense; thus, the large-scale long-term use for monitoring purposes to serve epidemiologic studies is currently out of reach (Kim et al. 2001a, 2001b). Although some experimental studies have been performed to investigate the oxidative activity of impinged particles (Cho et al. 2005; Li et al. 2003), most studies have used the same filter-based approaches as used in our study (Knaapen et al. 2001; Mudway et al. 2004; Schaumann et al. 2004; Shi et al. 2003b; Zielinski et al. 1999). The correlation and absolute difference between redox activity of freshly impinged PM and PM collected onto filters are currently under investigation.

Another question not addressed in our PM_2.5_-based study is the specific redox relevance of different-size fractions of PM. We and others have shown that, on a per unit mass basis, ultrafine particles are considerably more oxidatively active than are fine and coarse particle fractions (Cho et al. 2005; Li et al. 2003; Shi et al. 2003b). However, the larger fractions of PM_2.5_ remain important determinants of exposure to redox-active particles (per volume) given that the mass of ultrafine particles in ambient air is rather small (Cho et al. 2005). The mass is particularly large for the coarse fraction (2.5–10 μm), and health effects and redox activity in this fraction have also been shown to be significant (Brunekreef and Forsberg 2005; Shi et al. 2003b).

Finally, the way that traditional measures of air pollution are reported (mass/volume of air as a measure of air quality) contrasts with the units of oxidative properties [actual measures of particle quality (property/mass of particles)], which needs to be considered when interpreting our results.

We conclude that the approaches chosen in this study are valid to capture a toxicologically relevant feature of ambient PM. Findings should, however, not be generalized beyond the size fraction sampled in ECRHS or the fraction of PM that can be successfully recovered from filters. Studies that compare the currently employed methods to measure PM redox activity are warranted.

### Should these assays be used in epidemiologic studies?

Our results show that the oxidative properties of PM vary considerably across Europe and thus may explain part of the large heterogeneity in respiratory health symptoms reported in the ECRHS population (Janson et al. 2001). However, it appears inevitable from our data that these toxicologically relevant properties need to be measured given that no other PM characteristic served as a reliable surrogate. This was apparent both for short-term temporal patterns within cities and in the cross-community comparison of aggregate annual means. The filter-to-filter correlation between the ^•^OH formation and the ambient mass concentration of total PM or elemental constituents was not only low (Table 2) in many cases, but also heterogeneous across cities. This was also true for transition metals (Fe, Cu, V, Mn, and Ti), which are important sources of free radical formation (Aust et al. 2002; Prahalad et al. 1999). Factor analyses and other multivariate approaches may elucidate the specific multivariate determinants of PM redox activity. However, a city-by-city approach would be required given the large differences in redox-relevant PM characteristics across Europe (Götschi et al. 2005). Such analyses are beyond the scope of this article.

We also emphasize that the redox activity of PM-associated metals depends not only on the bulk concentration in the sample but also on bioavailability (for Fenton reactions), chemical speciation, and oxidation state (Shi et al. 2003a), which are not properly reflected in the elemental mass concentration derived by ED-XRF. This may partly explain the weak associations between metals and redox activity in our study. Correlations between absorbance—a proxy for elemental carbon—and redox activity were also weak, in contrast to recent findings in California showing a correlation coefficient of 0.89 between redox activity and elemental carbon (Cho et al. 2005). However, the latter study used an assay that is not sensitive to the metal content of PM, and pollution mixtures in southern California are likely to be significantly different from those in Europe; thus, comparability of these results is limited. Moreover, our measurements of light absorbance may in part be determined by the fraction of PM_2.5_ not recovered by our extraction (see above).

It is noteworthy that sulfur was the best correlate of redox activity in a few cities (Table 2) as well as across communities (annual means; Table 3). This underlies the importance of using surrogate measures when examining PM activity, because S content can not be simplistically related to established toxic pathways. The elemental S content of PM reflects both the concentration of secondary sulfate and primary metal sulfates derived from combustion processes in the samples. Secondary sulfate, derived from the oxidation of sulfur dioxide, represents the predominant form of S in PM_2.5_ but is unlikely to contribute significantly to the toxicity of the samples. Both human and animal challenge studies with sulfate salts have proven unable to duplicate any of the acute/chronic health effects related to PM exposure (Schlesinger et al. 1990; Utell et al. 1983). It has been argued, however, that sulfate may represent a proxy for the bioavailability of PM transition metals, because of both the strong association of these metals with acid aerosols and the capacity of sulfate to mobilize insoluble Fe from the surface of particles (Ghio et al. 1999). In addition to acting as possible ligands for Fe, sulfate can also modify transition-metal–catalyzed oxidative reactions *in vivo* by scavenging ^•^OH to yield less reactive inorganic radicals such as SO_4_^•−^ (De Laat et al. 2004).

The correlation between the oxidative properties and the other PM markers varied temporally at each of the sampling sites and regionally between cities, as has been shown previously (Shi et al. 2003a). This may explain differences observed in the toxicity profile across season (Salonen et al. 2004), location (Schaumann et al. 2004), or sources of PM (Greenwell et al. 2003) that have been reported in studies using surrogates rather than a direct measure of redox activity. The results of the two Antwerp locations (Table 2) also indicate that the heterogeneity of the correlations persists even within the same city, at two locations 11 km apart.

As shown in Table 3, none of the PM characteristics served as a proxy for the annual mean redox activity of the local PM. Particle oxidative activity was, on average, relatively low in some cities whereas levels of urban air pollution were high. Conversely, other locations appeared to have low mass concentrations, with high redox activity. This emphasizes that the oxidative properties of PM (per mass) are not related to ambient PM mass concentrations (per volume of air).

Several limitations and open questions need to be addressed before call for a large-scale use of these oxidative properties in epidemiologic studies. It is important to establish how PM oxidative activity measured at a central monitor relates personal PM exposures. Similarly, the relationship between activity measured at a single monitor as a marker of personal exposure and its relationship to spatial within-community and indoor/outdoor variation needs to be known. A recent French project and an extensive Dutch investigation showed that differences between personal and fixed site monitor concentrations vary considerably across PM constituents (or other pollutants), as well as across cities (Janssen et al. 2005; Meng et al. 2005; Nerriere et al. 2005; Oglesby et al. 2000; Sarnat et al. 2005). For example, although light absorbance and S concentrations showed rather high correlations with personal levels (Spearman rank correlations often > 0.9), outdoor measures were rather poor surrogates of personal exposure to calcium, Cu, or Si (usually ≪ 0.5) (Janssen et al. 2005). For pollution characteristics with very strong spatial gradients, such as reported for ultrafine particles or for numerous elements, such as Zn, Fe, Ni, or Cu (Zechmeister et al. 2005), in proximity to traffic arteries, a single monitor does not, therefore, appear to be an informative marker of personal exposure (Zhu et al. 2002). Given that these constituents are important determinants of redox activity, one has to expect large spatial gradients for oxidant activity, as well. This contention is supported by the data obtained from the two Antwerp monitoring sites (Table 1). Although average losses of AA from the synthetic RTLF varied 2.5 times across communities, AA depletion was, on average, two times larger in Antwerp City than in Antwerp South.

Therefore, study designs that rely on the assumption that the assigned measures reflect personal exposure of the study participants (e.g., panel studies, or cross-community comparisons of long-term effects) may need personal or at least home-based measurements to thoroughly investigate effects associated with PM oxidant activity. The lack of knowledge of spatial variation of oxidative properties limits the use of this novel information also in the ECRHS cross-community comparisons that are based on one single monitor per center.

In case of time-series studies, this may be less of a concern (Sheppard 2005; Sheppard et al. 2005; Zeger et al. 2000). Our data indicate that the temporal variability of ^•^OH formation and other traditionally used markers are of comparable size (see CV in Table 1). Thus, single-monitor daily measurements of PM ^•^OH generation may be of some value in time-series studies.

The application of these novel measures in the statistical model of epidemiologic investigations needs further clarification. Both ^•^OH-generating capacity and antioxidant depletion are expressed per standard mass of PM_2.5_, whereas mass and elemental content are characterized per volume of air as encountered by the lung under real-life conditions. The association between these radical-generating properties at a standard mass and ambient PM concentrations has been tested but varies between samples (Shi et al. 2003b). It would be useful to establish this function in a controlled toxicologic model. It is therefore interesting to note that a recent clinical study in which similar masses of PM_2.5_ were instilled into bronchi of healthy subjects showed different inflammatory responses that appeared to be related to the difference in the oxidative activity between the instilled PM samples (Schaumann et al. 2004). Redox activity of the standard mass may be used as an independent “exposure” term with or without PM ambient mass concentration as a coexposure. Alternatively, one may use an interaction term of PM and redox activity, assuming that the effect of air pollution on respiratory health depends both on the property of the inhaled particles (redox activity) and on the actual dose of such particles inhaled (PM concentration).

## Conclusion

In the present study, we have demonstrated that ^•^OH formation and antioxidant depletion by PM—both indicators of the capacity of inhaled PM to cause oxidative stress—vary substantially both temporally and regionally throughout Europe and are not well correlated with more readily available measurements of PM.

Use of these activity measures may be intriguing in epidemiologic studies but will require the establishment of appropriate methods for measuring PM redox activity and clarification of the relationship between ambient PM_2.5_ redox activity and the activity of the PM_2.5_ that actually reaches the subject’s lung (personal exposure).

## Figures and Tables

**Table 1:** PM_2.5_ characteristics measured at 20 locations in 19 European cities [annual means (coefficient of temporal variance)].

	No.	^•^OH	AA	GSH	PM_2.5_	Abs	S	Si	Al	Fe	Zn	Pb	Cu	Ti	As	Mn	V
Albacete, Spain	35	12,322 (0.26)	41.2 (0.22)	21.4 (0.68)	14.0 (0.37)	1.4 (0.29)	1,074 (0.46)	804 (1.12)	389 (1.13)	53 (0.96)	13.6 (1.78)	11.1 (1.16)	3.3 (1.98)	6.3 (1.05)	1.7 (0.87)	2.3 (0.59)	2.9 (0.55)
Antwerp City, Belgium	34	22,235 (0.51)	61.2 (0.19)	45.5 (0.39)	22.6 (0.98)	2.8 (0.47)	1,330 (0.67)	372 (0.74)	178 (0.77)	131 (0.73)	55.5 (1.30)	27.4 (1.10)	9.5 (1.22)	5.3 (0.71)	6.9 (1.56)	6.7 (0.88)	6.4 (0.89)
Antwerp South, Belgium	35	18,367 (0.40)	31.7 (0.28)	23.3 (1.23)	19.8 (0.85)	1.6 (0.67)	1,421 (0.56)	289 (1.03)	128 (0.82)	61 (0.72)	43.5 (1.26)	25.3 (1.02)	5.7 (1.91)	3.5 (0.82)	5.8 (1.30)	4.8 (0.76)	5.7 (0.91)
Barcelona, Spain	36	29,630 (0.38)	74.4 (0.14)	31.5 (0.73)	25.0 (0.54)	3.2 (0.51)	1,615 (0.48)	727 (0.57)	462 (1.06)	151 (0.53)	89.0 (0.76)	59.0 (0.90)	20.8 (0.90)	24.0 (1.40)	14.0 (0.90)	11.0 (1.07)	9.7 (0.76)
Basel, Switzerland	36	29,668 (0.45)	61.3 (0.13)	11.1 (0.89)	16.8 (0.68)	1.7 (0.44)	1,000 (0.63)	315 (0.60)	169 (0.95)	79 (0.53)	32.9 (0.82)	13.0 (0.86)	6.0 (0.87)	3.3 (0.58)	4.1 (1.09)	3.4 (0.67)	1.4 (0.67)
Erfurt, Germany	36	27,328 (0.32)	58.0 (0.21)	16.7 (1.07)	15.4 (0.69)	1.7 (0.63)	1,133 (0.68)	297 (0.70)	139 (0.67)	71 (0.75)	35.3 (1.12)	13.8 (1.25)	5.1 (1.04)	2.7 (0.77)	4.1 (1.05)	3.1 (0.70)	0.8 (0.81)
Galdakao, Spain	36	82,825 (0.83)	76.4 (0.07)	34.0 (0.21)	16.6 (0.51)	1.9 (0.43)	1,690 (0.83)	476 (0.73)	213 (0.76)	154 (0.61)	122.1 (0.78)	35.4 (0.64)	15.9 (0.76)	4.3 (0.73)	7.8 (0.83)	20.0 (0.76)	9.1 (1.01)
Goteborg, Sweden	35	24,817 (0.49)	53.3 (0.18)	12.9 (1.83)	14.0 (0.54)	1.1 (0.69)	1,044 (0.82)	221 (0.97)	100 (0.97)	56 (0.94)	17.5 (0.84)	5.9 (1.33)	4.1 (1.05)	2.2 (1.12)	2.4 (0.86)	3.1 (0.90)	4.2 (0.69)
Grenoble, France	36	27,139 (0.44)	68.4 (0.27)	28.9 (0.36)	19.1 (0.62)	2.6 (0.53)	875 (0.53)	1,322 (1.11)	270 (1.43)	125 (0.79)	170.0 (1.17)	23.9 (0.95)	18.2 (1.44)	6.1 (1.17)	5.6 (1.20)	10.1 (1.07)	3.2 (0.78)
Huelva, Spain	36	22,579 (0.50)	62.8 (0.31)	37.0 (0.57)	18.2 (0.51)	1.4 (0.53)	1,754 (0.90)	1,308 (0.64)	446 (0.63)	78 (0.48)	33.6 (1.16)	29.4 (1.22)	25.5 (1.33)	14.8 (1.94)	10.7 (1.44)	3.1 (0.59)	7.6 (0.81)
Ipswich, UK	31	16,543 (0.56)	46.2 (0.26)	11.0 (1.26)	16.9 (0.89)	1.3 (0.69)	975 (0.87)	181 (0.67)	142 (2.02)	39 (0.64)	27.1 (1.56)	25.7 (2.88)	6.1 (2.25)	6.0 (2.85)	8.4 (2.30)	3.5 (1.22)	5.5 (1.31)
Norwich, UK	34	16,276 (0.54)	33.5 (0.40)	27.0 (0.31)	18.3 (0.67)	1.7 (0.43)	1,082 (0.78)	207 (0.78)	116 (0.60)	47 (0.59)	18.0 (0.83)	15.6 (1.29)	3.9 (1.11)	3.1 (0.98)	4.9 (1.16)	2.6 (0.80)	5.8 (0.96)
Oviedo, Spain	35	25,966 (0.61)	51.9 (0.17)	43.1 (0.14)	16.7 (0.40)	2.1 (0.46)	1,280 (0.64)	878 (0.61)	509 (0.57)	149 (0.82)	33.2 (0.68)	23.4 (0.66)	9.4 (0.63)	8.1 (0.62)	6.0 (0.79)	7.8 (1.28)	5.8 (0.53)
Pavia, Italy	33	17,866 (0.52)	60.8 (0.22)	10.6 (0.86)	39.1 (0.66)	3.0 (0.35)	2,047 (0.51)	510 (0.66)	231 (0.63)	132 (0.39)	48.8 (0.86)	39.5 (0.62)	9.7 (0.56)	8.1 (0.92)	10.5 (0.60)	9.3 (0.98)	4.5 (0.52)
Paris, France	36	37,982 (0.33)	75.2 (0.11)	22.3 (0.39)	18.6 (0.57)	2.4 (0.38)	1,160 (0.60)	345 (0.86)	155 (0.88)	99 (0.54)	39.9 (0.73)	15.4 (0.90)	9.9 (0.84)	4.3 (0.64)	3.7 (0.77)	4.9 (1.15)	2.2 (0.66)
Reykjavik, Iceland	31	8,647 (0.59)	31.0 (0.30)	28.8 (0.59)	3.8 (0.72)	0.1 (1.74)	137 (1.14)	214 (1.18)	103 (1.25)	26 (1.11)	2.7 (0.88)	2.2 (2.74)	2.0 (1.18)	2.5 (1.48)	0.9 (1.31)	0.5 (1.22)	0.4 (6.21)
Tartu, Estonia	36	12,691 (0.48)	48.5 (0.13)	20.6 (0.96)	13.8 (0.55)	1.6 (0.47)	825 (0.60)	303 (1.39)	129 (1.35)	29 (1.05)	32.2 (0.64)	8.6 (0.94)	2.8 (1.30)	2.3 (1.19)	2.2 (0.97)	2.9 (1.05)	1.1 (0.77)
Turin, Italy	36	27,151 (0.57)	73.5 (0.13)	27.9 (0.62)	48.2 (0.59)	4.3 (0.31)	2,057 (0.55)	773 (0.55)	382 (0.53)	270 (0.48)	74.8 (0.75)	62.2 (0.52)	22.6 (0.63)	8.6 (0.52)	14.7 (0.53)	14.5 (0.79)	4.0 (0.56)
Umeå, Sweden	36	27,330 (0.40)	65.0 (0.19)	21.5 (0.75)	6.2 (0.46)	0.8 (0.61)	452 (0.78)	190 (0.99)	75 (0.82)	28 (0.73)	7.5 (0.86)	3.3 (1.84)	2.6 (1.75)	1.8 (1.06)	1.2 (0.76)	1.3 (0.80)	1.0 (1.20)
Uppsala, Sweden	36	33,307 (0.48)	73.6 (0.06)	12.4 (0.38)	11.3 (0.57)	1.1 (0.38)	840 (0.66)	238 (0.82)	101 (0.75)	53 (0.70)	15.9 (0.82)	5.9 (1.72)	3.9 (1.16)	1.9 (0.80)	1.9 (1.03)	2.0 (0.83)	1.7 (0.77)
Across centers	Mean	26,033	57.4	24.4	18.7	1.9	1,190	498.5	221.9	91.6	45.7	22.3	9.4	6.0	5.9	5.8	4.2
	SD	15,321	14.8	10.4	9.9	0.9	484	355.5	138.2	60.6	41.1	16.8	7.3	5.3	4.1	5.0	2.8
	Max/min	9.6	2.5	4.3	12.7	43.0	15.0	7.3	6.8	10.4	63.0	28.3	12.8	13.3	16.3	40.0	24.3

Abbreviations: Abs, absorbance; max, maximum; min, minimum. The last row presents the distribution of characteristics across all locations and the ratio between the annual mean in the city with the highest level and the lowest annual mean. Units: ^•^OH formation (of a mass standardized suspension; see “Materials and Methods”), arbitrary units; depletion rates of antioxidants (AA, GSH), percentages; absorbance, absorption coefficient/m; PM_2.5_ and total mass, mass concentrations in μg/m^3^; mass for all elements, ng/m^3^ (elements are sorted by mean mass concentration).

**Table 2:** Spearman rank temporal correlation between ^•^OH formation of PM_2.5_ and PM_2.5_ mass concentration, absorbance (Abs), and the mass concentration of the seven most abundant elements, by ECRHS location.

City	No.	PM_2.5_	Abs	S	Si	Al	Fe	Zn	Pb	Cu
Albacete	35	0.13	0.04	0.23	−0.02	0.02	0.14	0.01	0.21	0.04
Antwerp City^a^	33	−0.19	0.34	−0.28	0.17	0.13	0.23	0.21	0.39	0.31
Antwerp South^b^	34	−0.21	0.04	−0.25	0.14	0.16	0.14	0.24	0.11	0.03
Barcelona	36	0.61	0.49	0.57	0.37	0.34	0.39	0.22	0.43	0.41
Basel	36	−0.45	−0.06	−0.52	0.20	0.07	0.10	0.07	0.02	0.29
Erfurt	36	0.35	0.44	0.24	0.55	0.47	0.69	0.26	0.13	0.56
Galdakao	36	−0.08	0.11	−0.06	−0.15	−0.16	0.22	0.33	0.48	0.68
Goteborg	35	−0.36	0.01	−0.25	0.15	0.01	0.19	0.08	0.03	0.25
Grenoble	36	−0.02	0.28	0.33	0.10	−0.03	0.10	0.20	0.18	0.18
Huelva	36	0.34	0.20	0.45	0.36	0.13	0.17	0.48	0.41	0.43
Ipswich	31	0.34	0.30	0.50	0.16	0.15	0.36	0.11	0.19	0.03
Norwich	34	0.33	0.07	0.73	0.19	0.15	0.38	0.34	0.24	0.07
Oviedo	35	0.56	0.01	0.74	0.52	0.42	0.68	0.45	0.22	0.47
Paris	36	0.55	0.27	0.61	0.37	0.36	0.52	0.49	0.08	0.18
Pavia	33	−0.49	−0.38	−0.39	0.33	0.11	−0.05	0.04	−0.37	0.12
Reykjavik	31	−0.12	0.50	0.37	0.24	0.15	0.22	0.03	0.15	0.04
Tartu	36	0.06	−0.30	0.29	0.18	0.21	0.28	−0.41	−0.23	−0.03
Turin	36	−0.49	−0.12	−0.54	0.20	0.07	0.04	−0.28	−0.15	0.01
Umeå	36	0.05	0.28	−0.03	0.08	−0.02	0.21	0.04	−0.01	−0.03
Uppsala	36	0.06	0.21	0.01	0.22	0.06	0.54	0.33	0.14	0.22

a One outlier excluded (PM_2.5_ = 159 μg/m^3^).

b One outlier excluded (PM_2.5_ = 141 μg/m^3^).

**Table 3:** Cross-community Pearson correlations between the annual mean of ^•^OH formation, depletion rates of AA and GSH, PM_2.5_ mass concentration, light absorbance (Abs), and mass concentration of chemical elements on PM_2.5_.

	^•^OH	AA	GSH	PM_2.5_	Abs	S	Si	Al	Fe	Zn	Pb
AA	0.65	1									
GSH	0.18	0.08	1								
PM_2.5_	0.03	0.33	0.08	1							
Abs	0.16	0.49	0.28	0.93	1						
S	0.30	0.35	0.24	0.87	0.81	1					
Si	0.03	0.30	0.45	0.34	0.44	0.38	1				
Al	0.01	0.24	0.55	0.47	0.54	0.56	0.80	1			
Fe	0.45	0.59	0.41	0.85	0.90	0.78	0.45	0.58	1		
Zn	0.58	0.50	0.33	0.46	0.60	0.49	0.60	0.33	0.68	1	
Pb	0.30	0.45	0.36	0.88	0.89	0.85	0.43	0.60	0.94	0.63	1
Cu	0.39	0.60	0.49	0.63	0.69	0.72	0.74	0.74	0.76	0.67	0.80

Annual means are derived from six pooled bimonthly suspensions (AA, GSH) and 31–36 filters (all other PM measures), respectively (see Table 1 and “Materials and Methods”).
